# Micro-scale genetic structure and genetic variation of *Neotricula aperta* (Gastropoda: Pomatiopsidae), the intermediate host of *Schistosoma mekongi* (Digenea: Schistosomatidae) in Champasak Province, Laos

**DOI:** 10.1186/s41182-025-00775-9

**Published:** 2025-07-24

**Authors:** Naruemon Bunchom, Weerachai Saijuntha, Chairat Tantrawatpan, Wanchai Maleewong, Joseph Valencia, Takeshi Agatsuma, Virasack Bounavong, Philippe Buchy, Moritoshi Iwagami

**Affiliations:** 1 Department of Tropical Medicine and Malaria, Research Institute, Japan Institute for Health Security, Tokyo, Japan; 2 SATREPS Project for Parasitic Diseases (JICA/AMED), Vientiane, Laos; 3 Faculty of Medicine, and Biomedical Science Research Unit, Mahasarakham University, Mahasarakham, Thailand; 4 Center of Excellence in Biodiversity Research, Mahasarakham University, Mahasarakham, Thailand; 5 Division of Cell Biology, Department of Preclinical Sciences, Faculty of Medicine, and Center of Excellence in Stem Cell Research and Innovation, Thammasat University, Rangsit Campus, Pathum Thani, Thailand; 6 Department of Parasitology, Faculty of Medicine, and Mekong Health Science Research Institute, Khon Kaen University, Khon Kaen, Thailand; 7 Graduate School of Health Sciences, Niigata University, Niigata, Japan; 8 Immunology Department, Research Institute for Tropical Medicine, Philippine Department of Health, Muntinlupa, Metro Manila Philippines; 9 Department of Environmental Health Sciences, Kochi Medical School, Nankoku, Kochi Japan; 10 Center of Malariology, Parasitology and Entomology, Ministry of Health, Vientiane, Laos; 11 Institut Pasteur du Laos, Ministry of Health, Vientiane, Laos

**Keywords:** *Neotricula aperta*, Schistosomiasis, Elimination, Transmission, Genetic diversity, Unique genetic differences, Laos

## Abstract

**Background:**

*Neotricula aperta*, a freshwater snail found in the Mekong River, serves as the intermediate host of the blood fluke *Schistosoma mekongi*, the causative agent of schistosomiasis mekongi in Cambodia and Laos. Understanding the genetic diversity, population structure of this snail in relation to its geographical distribution is crucial for a comprehensive understanding of disease transmission. In this study, we investigated the genetic diversity, and genetic structure of *N. aperta* in Champasak Province, Laos.

**Methods:**

A total of 80 *N. aperta* snails were collected from 13 various localities across five villages in Khong and Mounlapamok districts in Champasak Province, Laos in May 2024. Species of snails were initially identified based on morphology and subsequently confirmed by DNA barcoding. Molecular analyses were conducted using specific primers to amplify two mitochondrial DNA genes, namely cytochrome *c* oxidase subunit 1 (*cox1*) and 16S ribosomal RNA (16S rRNA), in order to assess the genetic diversity of *N. aperta* populations.

**Results:**

Based on *cox1* sequences, the overall haplotype diversity was 0.996, while the overall nucleotide diversity was 0.049. For the 16S rRNA marker, the overall haplotype diversity was 0.911, and the overall nucleotide diversity was 0.015. Analysis of molecular variance (AMOVA) confirmed significant genetic differentiation (*P*-value < 0.05) among populations at different spatial scales, including villages, catchments, and countries. This genetic structure likely reflects limited gene flow among snail populations, potentially due to geographical barriers. Although local environmental factors may also contribute to differentiation, the current genetic data are insufficient to distinguish between geographic isolation and adaptive divergence. Further ecological and functional investigations will be needed to determine whether adaptive processes are influencing population structure.

**Conclusions:**

The genetic divergence of *N. aperta* observed in this study indicates a high level of genetic differentiation both among and within populations. This pattern suggests that *N. aperta* is possibly undergoing localized adaptation or that barriers to gene flow, such as physical, ecological, or behavioral factors, are promoting the accumulation of genetic differences. Micro-scale genetic structuring within populations may be driven by microhabitats or small-scale ecological gradients, while limited dispersal, localized mating preferences, or other behavioral traits may contribute to differentiation among populations.

**Supplementary Information:**

The online version contains supplementary material available at 10.1186/s41182-025-00775-9.

## Background

**Fig. 1:**
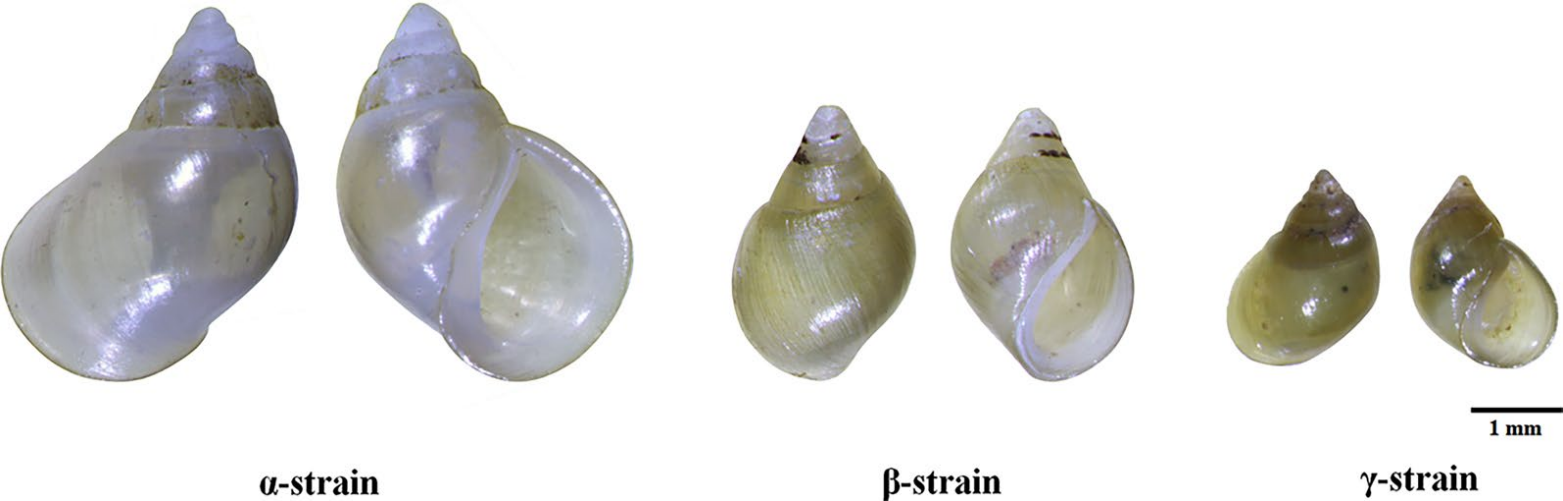
Shell characteristics of *Neotricula aperta* from Southeast Asia

*Neotricula aperta*, a freshwater snail, functions as the exclusive intermediate host of *Schistosoma mekongi*, the etiological agent of schistosomiasis mekongi, a significant public health concern endemic to the Mekong River basin, encompassing regions of Thailand, Cambodia, and Laos [1–5]. Three morphologically distinct groups of *N. aperta*—designated α, β, and γ—have been identified based on variation in morphology (e.g., shells (Fig. 1), pigment patterns, and reproductive systems), histology, ecology, distribution, systematics, and evolutionary relationships [6]. However, genetic analyses have revealed no significant differentiation among them [1, 4]. The γ-strain of *N. aperta* is of particular epidemiological importance in natural habitats, as it serves as the principal intermediate host of *S. mekongi*, the parasitic trematode responsible for schistosomiasis mekongi [7]. *Neotricula aperta* typically inhabits freshwater environments, including streams, ponds, and irrigation channels [8, 9]. Human infection occurs through contact with water contaminated by cercariae of *S. mekongi*. The distribution and abundance of *N. aperta* are influenced by specific ecological factors, including water quality, shallow depths (0.5–3.0 m), and alkaline conditions (pH > 7.5) [3]. Additionally, paratenic or potential reservoir hosts including pigs and dogs can also become infected with *S. mekongi*, potentially contributing to the persistence and transmission of this parasite [10, 11].

In 2017, the World Health Organization (WHO) launched a regional strategy to accelerate the elimination of schistosomiasis in the Western Pacific Region, targeting endemic countries such as Cambodia and Laos [12]. This initiative supports the global objective of eliminating schistosomiasis as a public health problem by 2025 and interrupting transmission by 2030 in specific regions. A key focus of this strategy is the elimination of *S. mekongi*, a species endemic to the Mekong River basin [13]. Effective control of schistosomiasis critically depends on interrupting the parasite’s transmission cycle, particularly through the management of the snail intermediate host. This strategy has demonstrated success in other settings, for instance, *Schistosoma japonicum* was eliminated from Japan primarily through sustained efforts to reduce its snail host populations [9, 14]. Similarly, the control and prevention of the liver fluke *Opisthorchis viverrini* in Thailand's Lawa model tended to be achieved by targeting and eliminating its snail intermediate hosts in endemic areas [15].

Understanding the genetic diversity and population structure of *N. aperta* is vital to unraveling the dynamics of disease transmission [1–5]. Molecular tools, particularly mitochondrial DNA markers, have been widely used to explore the genetic structure of *N. aperta* populations [1–5]. Patterns of genetic variation within and among populations can offer insights into how *N. aperta* adapts to localized environmental conditions, such as variations in water quality, habitat type, and levels of exposure to *S. mekongi* infections [5]. Natural selection may favor genetic variants that confer increased resistance to parasitic infection or tolerance to specific ecological conditions, leading to adaptive divergence among populations. However, evolutionary processes such as genetic drift, inbreeding, and habitat fragmentation—particularly in small or isolated populations—can reduce genetic diversity. This loss of diversity may constrain the potential for adaptive evolution, limiting the species' ability to respond to environmental change or parasitic pressures [5].

Genetic variation in *N. aperta* may influence susceptibility to *S. mekongi*, with some populations potentially evolving immune or behavioral adaptations through localized coevolution [5]. In turn, *S. mekongi* may adapt to specific snail genotypes, resulting in a complex host–parasite dynamic. Understanding the genetic structure of *N. aperta* is therefore crucial for predicting transmission patterns and designing targeted control strategies. Populations with higher susceptibility may require more intensive interventions, such as molluscicide application or habitat modification. Moreover, if resistant snail strains emerge, control approaches can be adjusted accordingly. Recent studies using mitochondrial DNA markers have begun to reveal substantial genetic diversity and population structuring across Laos, Cambodia, and Thailand [1–5]. For instance, the highly susceptible γ-strain of *N. aperta*, originally isolated from Laos, has been linked to endemic transmission in Cambodia [16].

The population structure of *N. aperta* is often shaped by geographic barriers such as rivers, mountains, and isolated wetlands, which can limit gene flow and lead to genetic differentiation among populations [2, 5]. Snail populations in separate river basins or fragmented habitats may exhibit distinct genetic signatures, reflecting their level of isolation [2]. Analyzing the spatial distribution of genetic variation alongside environmental variables and *S. mekongi* prevalence can offer valuable insights into transmission dynamics [17]. Mitochondrial DNA markers are frequently used to examine phylogenetic relationships, quantify genetic diversity, and infer gene flow across landscapes. Integrating genetic, environmental, and epidemiological data enhances our understanding of host–parasite interactions and supports the development of targeted schistosomiasis control strategies.

In this cross-sectional study, we employed two mitochondrial DNA markers, namely cytochrome* c* oxidase subunit 1 (*cox1*) and 16S ribosomal RNA (16S rRNA) to investigate the genetic diversity and population structure of *N. aperta*. These markers are widely used in population genetics due to their high amplification success, extensive reference data, and utility in distinguishing closely related taxa. The *cox1* gene, commonly used in DNA barcoding, offers high resolution for detecting genetic variation among closely related populations. In contrast, the 16S rRNA gene is more conserved and is often used to infer deeper phylogenetic relationships and assist in taxonomic classification. Together, these markers provide a robust framework for examining genetic differentiation and the evolutionary dynamics of *N. aperta* across various localities in the Khong and Mounlapamok districts of Champasak Province, Laos.

## Materials and methods

### Specimen collection and identification

**Fig. 2:**
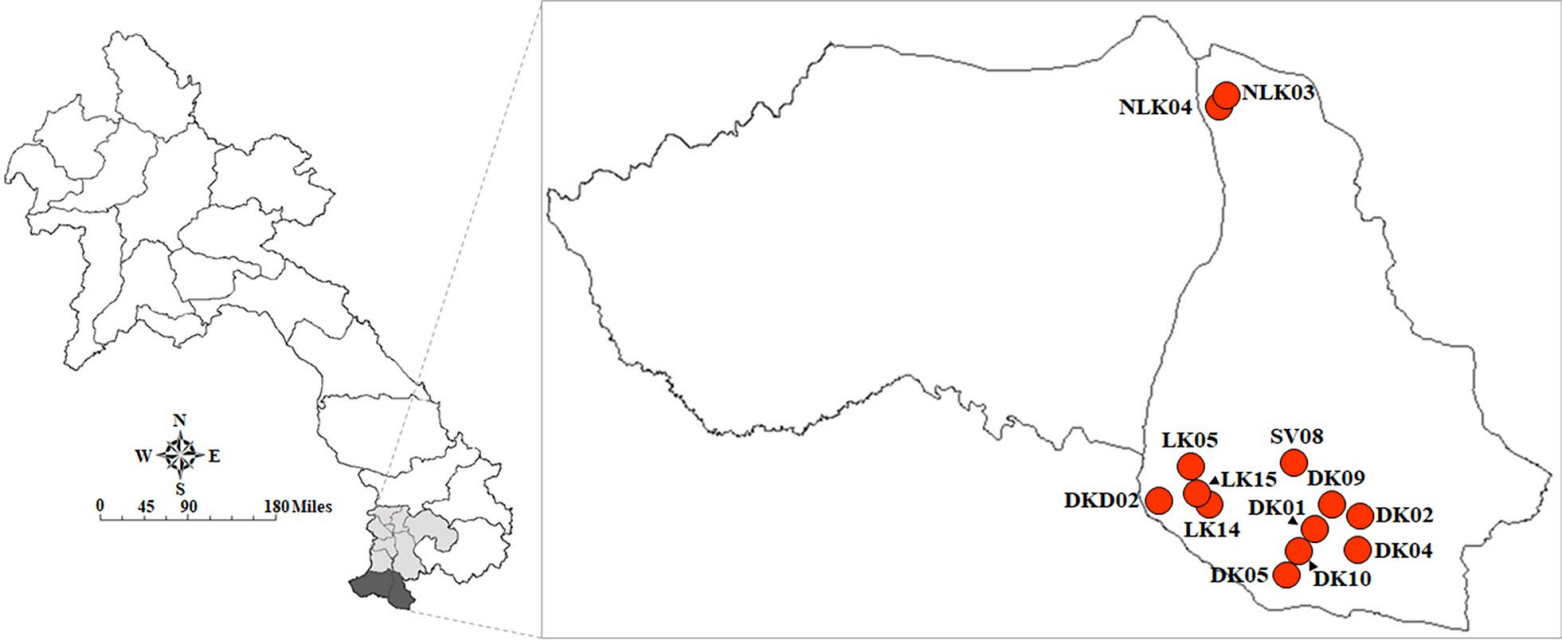
Sampling localities of *Neotricula aperta* in the Khong and Mounlapamok districts of Champasak Province, Laos. DKD: Don Kadent; DK: Don Khone; LK: Longkang; SV: Somven-ok; NLK: Nangloy Kang

**Fig. 3:**
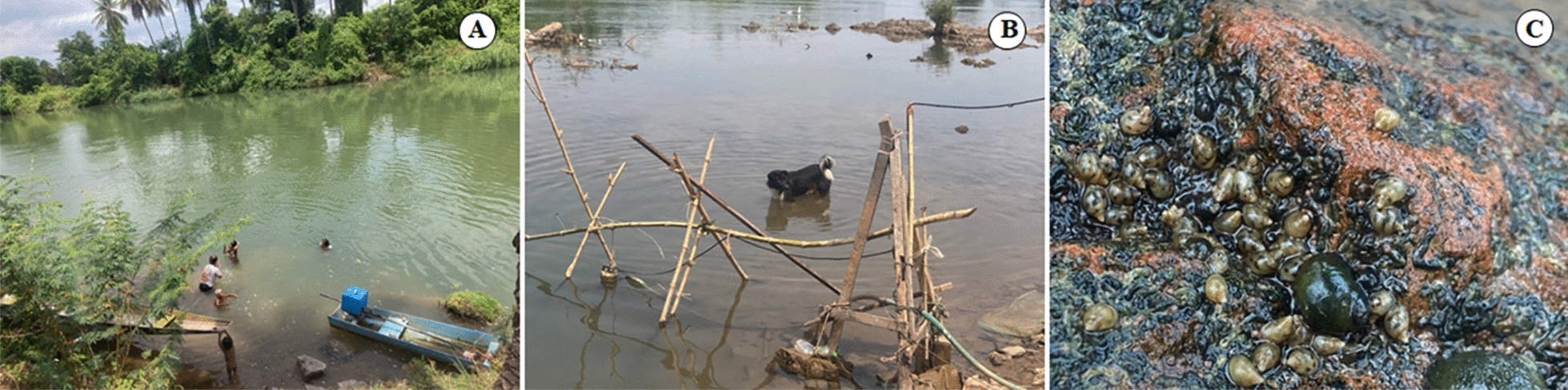
Natural habitats of *Neotricula aperta* in the Mekong River: **A** humans (definitive hosts) bathing in the Mekong River; **B** a dog soaking in the Mekong River; **C** *N. aperta* population on natural bedrock

*Neotricula aperta* collection campaigns were organized quarterly: in February, May, August, and October 2024. However, snail specimens were only found in May. The collection campaigns involved 13 localities across five villages along the Mekong River, located in the Khong and Mounlapamok districts of Champasak Province, southern Laos (Supplementary Table 1; Figs. 2 and 3). Snails were collected by hand picking and preserved in 80% (v/v) ethanol until further use. Species identification of *N. aperta* was based on morphological characteristics [6].

### Molecular study

DNA was extracted from the entire body of each individual snail using the DNeasy Blood and Tissue Kit (QIAGEN, Germany), following the manufacturer's protocol for molecular studies. The primers LCO1490 and HCO2198 [18] were used to amplify an approximately 630 bp fragment of the *cox1* gene. An approximately 490 bp fragment of the 16S rRNA gene was amplified using primers 16Sar-L and 16Sbr-H [19]. PCR reactions and conditions for both genes followed the protocols described by Bunchom et al. [20, 21]. PCR products were stained with 3X Loading Buffer Orange G (Nippon Gene Co. Ltd., Japan) and examined by electrophoresis on a 2% agarose gel. Successfully amplified products were purified using the FastGene™ Gel/PCR Extraction Kit (Nippon Genetics Co. Ltd., Japan), following the manufacturer’s instructions. DNA sequencing was carried out using the Applied Biosystems 3130xl Genetic Analyzer (Hitachi Co. Ltd., Japan).

### Data analysis

In total, 80 *cox1* sequences (accession nos. PV592087-PV592166) and 80 16S rRNA sequences (accession nos. PV592171–PV592250) were obtained from representative samples identified by morphological characteristics as *N. aperta* γ-strain. Additionally, a total of 448 *cox1* sequences were retrieved from GenBank and included in the data analyses (Supplementary Table 1). These comprised 433 sequences of the *Neotricula aperta* γ-strain [1, 4, 5], 12 sequences of the β-strain [1, 4], and three sequences of the α-strain [1]. All sequences were aligned using BioEdit version 7.2.6 [22]. To improve alignment quality, gaps present in the 5′-end (positions 1–20 bp) of the 16S rRNA sequences were trimmed prior to analysis. A total of 251 sequences of the *N. aperta* γ-strain were included in the alignment, comprising 80 newly obtained sequences from this study and 171 sequences retrieved from GenBank. Two genetic diversity indices, namely haplotype diversity (Hd) and nucleotide diversity (π) were calculated across 13 localities using DnaSP v5.10.01 [23]. Pairwise genetic differentiation (Φ_ST_) was calculated for sequences from five villages, Don Kadent, Don Khone, Longkang, Somven-ok, and Nangloy Kang, and used in the analysis of molecular variance (AMOVA) with Arlequin v3.5.2.2 [24]. Phylogenetic relationships were inferred using neighbor-joining (NJ) and maximum likelihood (ML) methods based on the GTR + G + I model, with 1000 bootstrap replicates, implemented in MEGA XI [25]. Genetic relationships among *cox1* and 16S rRNA haplotypes were further analyzed using a median-joining network (MJN) [26]; constructed with Network v5.0.1.1 (https://www.fluxus-engineering.com).

## Results

A total of 80 *N. aperta* samples (Supplementary Table 1), including those examined in this study and retrieved from GenBank, were used for molecular analyses. Based on the *cox1* sequences from 13 sampling sites in Champasak Province, the overall haplotype diversity was 0.996, ranging from 0.929 to 1.000, while the overall nucleotide diversity was 0.049, ranging from 0.003 to 0.050 (Table 1). For the 16S rRNA gene, the overall haplotype diversity was 0.911, with a range from 0.250 to 1.000, and the overall nucleotide diversity was 0.015, ranging from 0.001 to 0.016 (Table 1). Pairwise genetic differentiation (Φ_ST_) analysis revealed significant genetic structuring among sub-populations (*P* < 0.05), with Φ_ST_ values ranging from 0.011 to 0.989 for *cox1* and from 0.014 to 0.986 for 16S rRNA (Table 2).

**Table 1:** Diversity indices of *cox1* and 16S rRNA sequences in *Neotricula aperta* populations from 13 localities in Champasak Province, Laos

Populations	*cox1*						16S rRNA					
	*N*	*s*	*H*	*Uh*	Hd ± SD	π ± SD	*N*	*s*	*H*	*Uh*	Hd ± SD	π ± SD
DKD02	4	0	1	0	0.000 ± 0.000	0.000 ± 0.000	4	0	0	0	0.000 ± 0.000	0.000 ± 0.000
DK01	8	15	8	8	1.000 ± 0.063	0.008 ± 0.001	8	1	2	1	0.250 ± 0.180	0.001 ± 0.000
DK02	3	11	3	2	1.000 ± 0.272	0.012 ± 0.004	3	2	3	1	1.000 ± 0.272	0.003 ± 0.001
DK04	10	34	10	9	1.000 ± 0.045	0.014 ± 0.003	10	11	7	3	0.867 ± 0.107	0.005 ± 0.001
DK05	6	8	5	4	0.933 ± 0.122	0.005 ± 0.001	6	3	4	0	0.867 ± 0.129	0.002 ± 0.001
DK09	4	12	4	4	1.000 ± 0.177	0.010 ± 0.002	4	0	0	0	0.000 ± 0.000	0.000 ± 0.000
DK10	16	94	15	9	0.992 ± 0.025	0.050 ± 0.008	16	19	7	2	0.792 ± 0.076	0.016 ± 0.002
LK05	4	23	4	3	1.000 ± 0.177	0.019 ± 0.006	4	3	4	1	1.000 ± 0.177	0.003 ± 0.001
LK14	2	9	2	1	1.000 ± 0.005	0.014 ± 0.007	2	1	2	1	1.000 ± 0.500	0.002 ± 0.001
LK15	8	15	6	4	0.929 ± 0.084	0.010 ± 0.001	8	3	4	0	0.857 ± 0.082	0.003 ± 0.000
SV08	6	14	6	5	1.000 ± 0.096	0.007 ± 0.001	6	4	4	3	0.800 ± 0.172	0.003 ± 0.001
NLK03	7	7	6	4	0.952 ± 0.096	0.005 ± 0.001	7	4	5	2	0.857 ± 0.137	0.003 ± 0.001
NLK04	2	2	2	1	1.000 ± 0.005	0.003 ± 0.002	2	1	2	0	1.000 ± 0.005	0.002 ± 0.001
Total	80	129	70	54	0.996 ± 0.003	0.049 ± 0.004	80	36	26	14	0.911 ± 0.016	0.015 ± 0.001

*N*: number of *Neotricula aperta* examined; *s*: number of polymorphic sites;* H*: number of haplotypes; *Uh*: unique haplotypes; Hd: haplotype

Diversity; π: nucleotide diversity

**Table 2:** Pairwise genetic differentiation (Φ_ST_) among *Neotricula aperta* populations from 13 different localities in Champasak Province, Laos, based on *cox1* (lower triangle) and 16S rRNA (upper triangle) sequences

Populations	DKD02	DK01	DK02	DK04	DK05	DK09	DK10	LK05	LK14	LK15	SV08	NLK03	NLK04
DKD02	-	0.970**	0.961	0.882**	0.950*	1.000*	0.409*	0.947*	0.982	0.938*	0.941**	0.936*	0.984*
DK01	0.877**	-	0.957*	0.886**	0.950**	0.986**	0.516**	0.948**	0.971*	0.937**	0.941**	0.937**	0.974*
DK02	0.947	0.906**	-	0.041	0.376	0.724*	0.099	0.000	0.195	0.080	0.077	0.887*	0.894
DK04	0.878**	0.875**	0.112	-	0.040	0.196*	0.193	0.000	0.000	0.000	0.000	0.837**	0.804**
DK05	0.964**	0.925**	0.422*	0.011	-	0.014	0.174	0.240*	0.236	0.121	0.214	0.895**	0.899
DK09	0.939*	0.902**	0.150	0.000	0.095	-	0.158	0.571	0.822	0.366	0.529*	0.925*	0.978
DK10	0.406**	0.531**	0.123	0.162*	0.139*	0.094	-	0.136	0.014	0.193	0.169	0.579**	0.477**
LK05	0.891*	0.882**	0.000	0.067	0.301*	0.137	0.124	-	0.000	0.000	0.000	0.881**	0.878*
LK14	0.957*	0.904*	0.000	0.000	0.259*	0.000	0.000	0.000	-	0.036	0.000	0.883*	0.905
LK15	0.922**	0.901**	0.201*	0.119**	0.218**	0.000	0.181*	0.202*	0.000	-	0.019	0.890**	0.889*
SV08	0.946*	0.916**	0.045	0.095*	0.361**	0.264*	0.185*	0.023	0.060	0.327**	-	0.883**	0.883
NLK03	0.951**	0.911**	0.912**	0.874**	0.937**	0.913**	0.562**	0.876**	0.916*	0.908**	0.922**	-	0.000
NLK04	0.989	0.907*	0.890	0.847*	0.945	0.895	0.454*	0.819	0.891	0.894*	0.918*	0.149	-

*P*-value calculated at a confidence level of 95% * < 0.05 and ** < 0.001

**Fig. 4:**
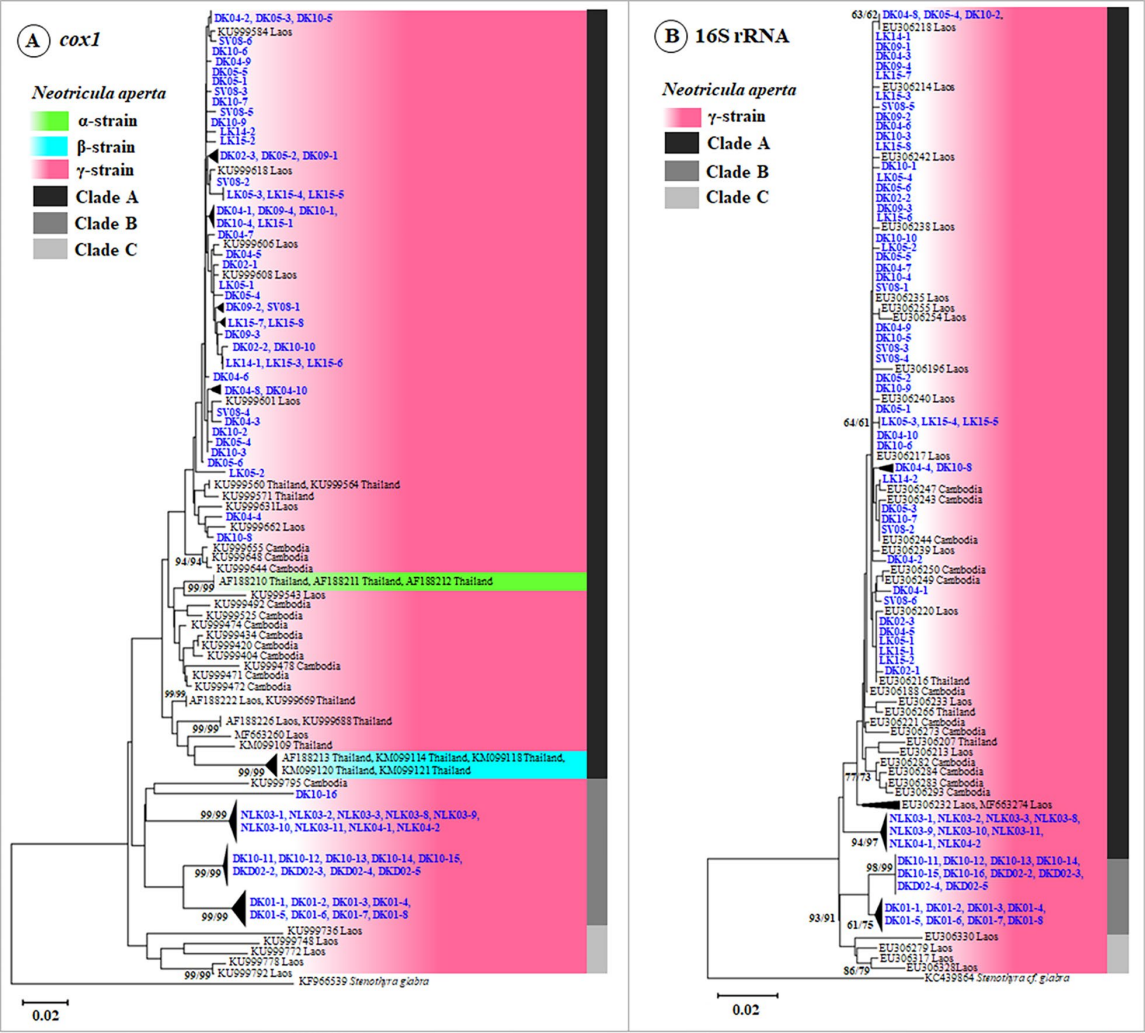
Phylogenetic tree of *Neotricula aperta* from Khong and Mounlapamok districts in Champasak Province, Laos: **A** *cox1* sequences; **B** 16S rRNA sequences. Neighbor-joining (NJ) and maximum likelihood (ML) methods, with bootstrap support values based on 1000 replications were indicated at the branch nodes

The phylogenetic analysis revealed that *N. aperta* was classified into three clades (A–C). Sequences obtained in this study clustered with clades A and B based on *cox1* and 16S rRNA sequences (Fig. 4). The *cox1* phylogenetic tree showed that clade A included *N. aperta* α-, β-, and γ-strains from Thailand, Cambodia, and Laos, including 53 sequences obtained in this study, and was the largest clade. Clade B comprised *N. aperta* γ-strain sequences from Laos (n = 27) obtained in this study, along with one sequence from Cambodia. Clade C included *N. aperta* γ-strain sequences from Laos. Since 16S rRNA sequences of *N. aperta* α- and β-strains are not available in GenBank, only the γ-strain was represented in the 16S rRNA phylogenetic analysis. The phylogenetic tree based on 16S rRNA also revealed three clades (A–C), consistent with the *cox1* phylogeny. Clade A was the largest, comprising 62 sequences of *N. aperta* γ-strain from Thailand, Cambodia, and Laos (obtained in this study). Clade B consisted of 18 sequences from Laos, while clade C included additional sequences from Laos, representing a distinct lineage within the γ-strain.

**Fig. 5:**
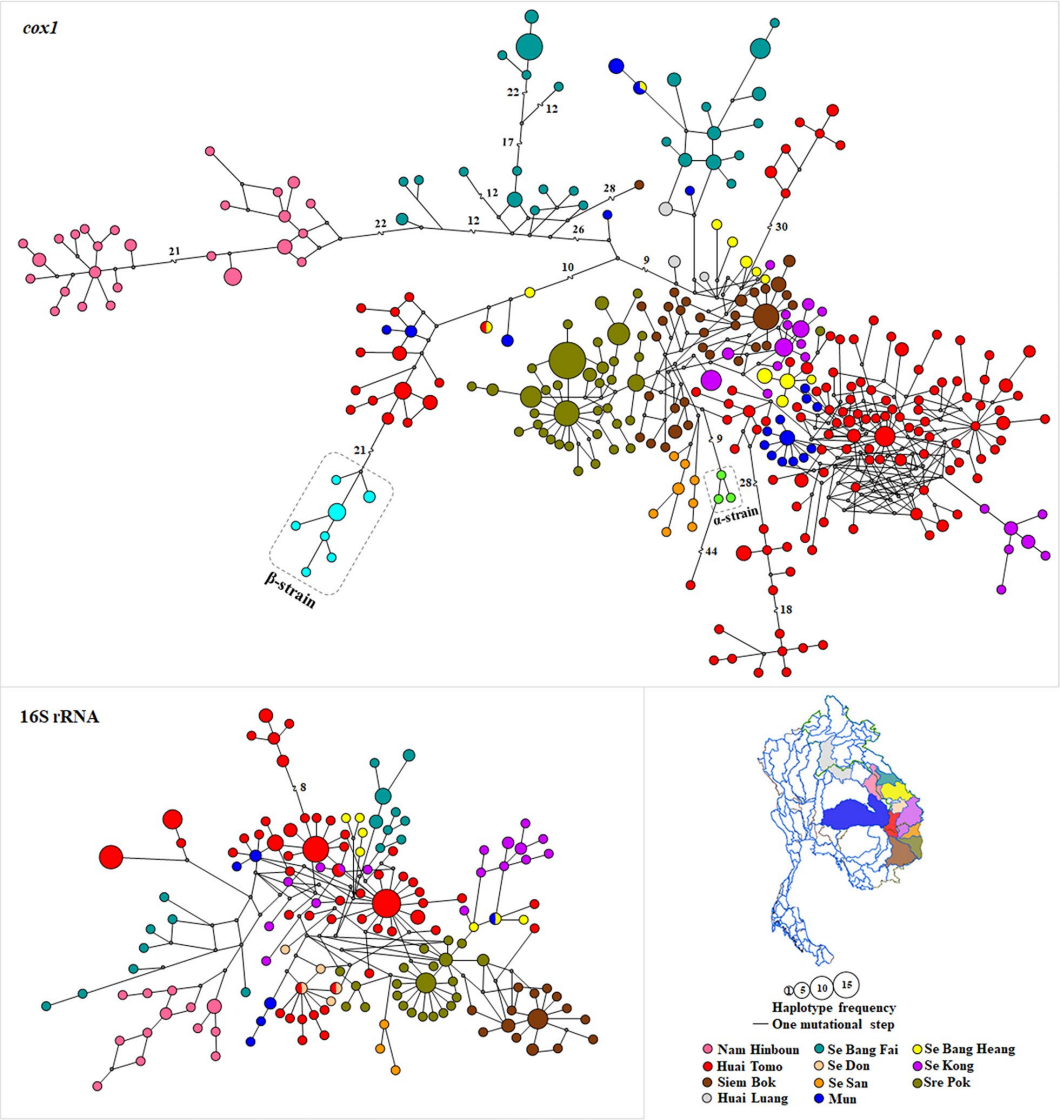
Median-joining network (MJN) based on mitochondrial *cox1* and 16S rRNA sequences of *Neotricula aperta*. Haplotypes are represented by circles. Groups are defined by sub-catchments in the Mekong River

The haplotype network analysis, based on 528 *cox1* sequences (80 obtained in this study and 448 retrieved from GenBank), revealed 320 haplotypes, of which 318 were unique to individual catchments. This high level of haplotype uniqueness indicates restricted gene flow and limited snail distribution between catchments (Fig. 5 and Table 3). A common haplotype was found in 20 samples from the Sre Pok catchment in Cambodia. Only two haplotypes were shared between different catchments: one was shared by two samples, one from Huai Tomo and one from Se Bang Heang. The other was shared by three samples, two from the Mun catchment and one from Se Bang Heang. Similarly, analysis of 251 16S rRNA sequences (80 obtained in this study and 171 retrieved from GenBank) identified 154 haplotypes. The 16S rRNA haplotype network revealed clear genetic structuring among catchment areas, consistent with limited gene flow between populations. Notably, only four haplotypes were shared across different catchments: two haplotypes were shared between Huai Tomo and Se Don (each represented by one sample), one haplotype was shared between the Mun and Se Bang Heang catchments (one sample each), and one haplotype was shared between Se Kong (one sample) and Huai Tomo (two samples) (Fig. 5 and Table 3). These limited haplotype overlaps further support the geographic structuring and potential local adaptation of *N. aperta* populations.

**Table 3:** Analysis of molecular variance (AMOVA) based on *cox1* and 16S rRNA sequences of *Neotricula aperta* γ-strain populations

Source of variation	*cox1*					16S rRNA				
	df	Ss	Vc	% variation	Fixation index	df	Ss	Vc	% variation	Fixation index
Group defined by 5 different villages (*N* = 80) both genes: *cox1* and 16S rRNA [Don Kadent (*N* = 4), Don Khone (*N* = 47), Longkang (*N* = 14), Somven-ok (*N* = 6), and Nangloy Kang (*N* = 9)]										
Among groups	4	370.514	4.991	35.37	*F*_CT_ = 0.517**	4	121.540	1.658	36.53	*F*_CT_ = 0.527**
Among populations within groups	8	260.923	4.714	33.41	*F*_SC_ = 0.688**	8	83.545	1.517	33.42	*F*_SC_ = 0.670**
Within populations	67	295.126	4.405	31.22	*F*_ST_ = 0.354*	67	91.365	1.364	30.04	*F*_ST_ = 0.365*
Group defined by 10 different catchments [*cox1* (*N* = 513): Huai Tomo (*N* = 162), Se Kong (*N* = 41), Nam Hinboun (*N* = 39), Se Bang Heang (*N* = 16), Se Bang Fai (*N* = 59), Siem Bok (*N* = 51), Se San (*N* = 9), Sre Pok (*N* = 93), Mun (*N* = 37), Huai Luang (*N* = 6)] [16S rRNA (*N* = 251): Huai Tomo (*N* = 112), Se Kong (*N* = 19), Nam Hinboun (*N* = 17), Se Bang Heang (*N* = 7), Se Bang Fai (*N* = 25), Se Don (*N* = 5), Siem Bok (*N* = 24), Se San (*N* = 3), Sre Pok (*N* = 31), Mun (*N* = 8)]										
Among groups	9	2667.624	4.568	32.64	*F*_CT_ = 0.604**	9	397.454	1.518	32.17	*F*_CT_ = 0.557**
Among populations within groups	37	2285.873	5.695	40.69	*F*_SC_ = 0.733**	35	389.406	1.784	37.80	*F*_SC_ = 0.670**
Within populations	467	1743.309	3.733	26.67	*F*_ST_ = 0.326**	207	293.414	1.417	30.03	*F*_ST_ = 0.322**
Group defined by 3 different countries [*cox1* (*N* = 513): Laos (*N* = 287), Cambodia (*N* = 161), Thailand (*N* = 65)]; [16S rRNA (*N* = 251): Laos (*N* = 188), Cambodia (*N* = 55), Thailand (*N* = 8)]										
Among groups	2	815.295	1.930	13.67	*F*_CT_ = 0.694**	2	88.512	0.432	9.38	*F*_CT_ = 0.660**
Among populations within groups	44	4138.201	8.456	59.89	*F*_SC_ = 0.736**	42	698.348	2.754	59.83	*F*_SC_ = 0.692**
Within populations	467	1743.309	3.733	26.44	*F*_ST_ = 0.137**	207	293.414	1.417	30.79	*F*_ST_ = 0.094*

*P*-value calculated at a confidence level of 95% * < 0.05 and 99%** < 0.001; df: degree of freedom; Ss: sum of squares; Vc: variance components

**Fig. 6:**
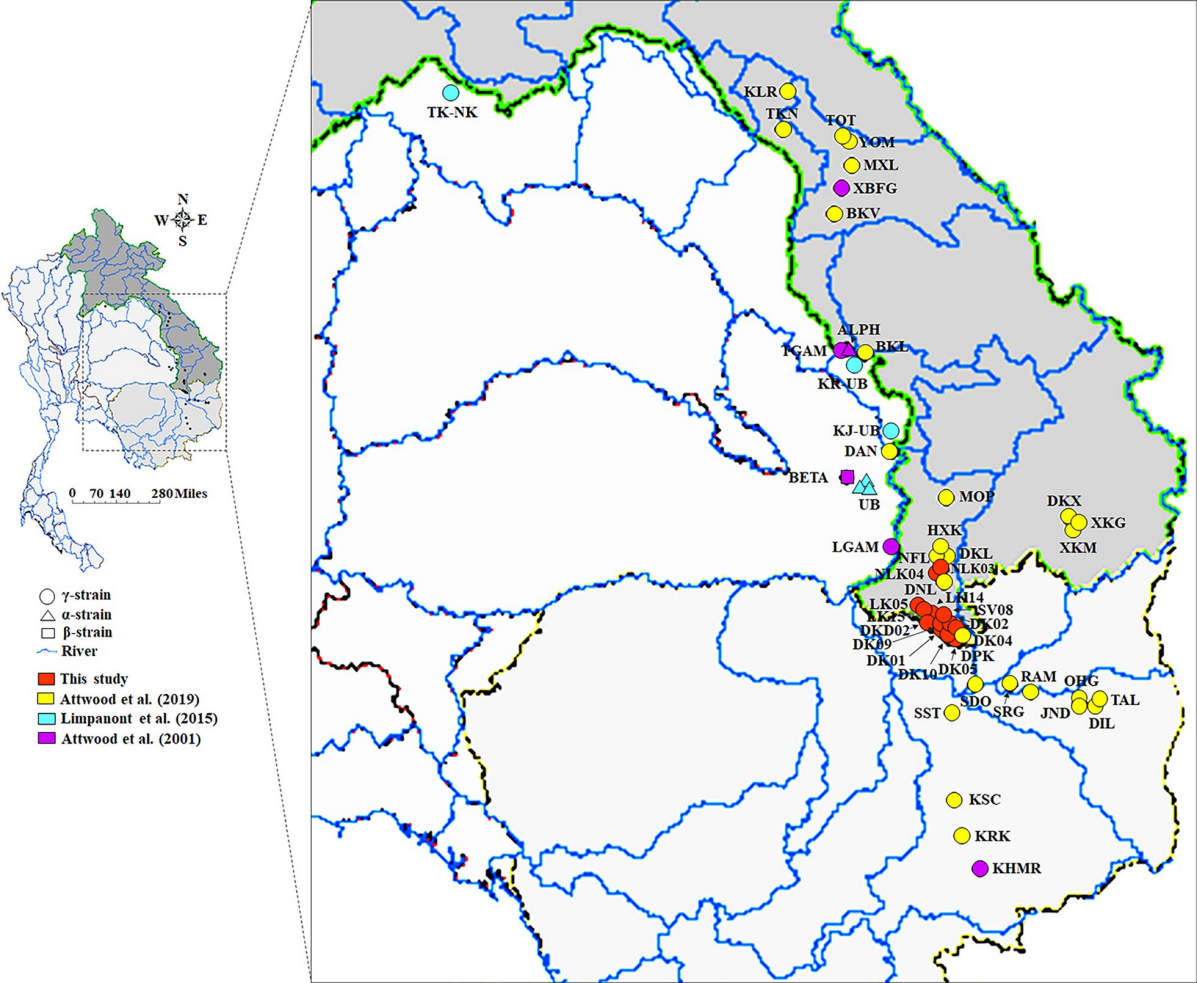
Geographical distribution of *Neotricula aperta* based on *cox1* sequences from Southeast Asia in Thailand, Cambodia, and Laos

The AMOVA based on *cox1* and 16S rRNA sequences revealed significant genetic differentiation among the five villages. Genetic variation was significant among groups (*F*_CT_ = 0.517, *P* < 0.001 for the *cox1* and *F*_CT_ = 0.527, *P* < 0.001 for 16S rRNA), among populations within groups (*F*_SC_ = 0.688, *P* < 0.001 for the *cox1* and *F*_SC_ = 0.670, *P* < 0.001 for 16S rRNA), and within populations (*F*_ST_ = 0.354, *P* < 0.05 for the *cox1* and *F*_ST_ = 0.365, *P* < 0.05) (Table 3). Genetic groupings corresponded closely with the five sampled villages, revealing significant genetic differentiation among *Neotricula aperta* populations within Champasak Province (Table 3). Additionally, these groupings were also structured by river catchments and geographical boundaries in each country, further supporting substantial genetic differentiation among *N. aperta* populations across Laos, Cambodia, and Thailand (Table 3; Fig. 6).

## Discussion

This study investigates the genetic structure of *N. aperta* populations from Khong and Mounlapamok districts in Champasak Province, Laos, highlighting significant micro-scale impacts on local ecosystems. The fragmentation of populations, reduction in gene flow, and potential changes in habitat have likely contributed to these effects [5]. Our findings revealed that the genetic structure of *N. aperta* became more fragmented and differentiated into smaller sub-populations across micro-scale geographical distances following the construction of a dam. This suggests that the dam acted as a barrier, limiting gene flow between populations and promoting independent evolutionary paths over time. For example, the construction of the Pak-Mun dam, which obstructed the natural flow regime of the Mekong River even before full impoundment, has been associated with potential impacts related to climate change and dam-induced alterations in flow, affecting *N. aperta* habitat characteristics such as sediment drift and water quality [8, 27]. Sediment architecture and water quality are key determinants of the aquatic food web components that support *N. aperta* populations, particularly macrophytes, microorganisms, and macroinvertebrates. On Khong Island, the habitat for *N. aperta*, as described in the Population-Based Control Model (PBCM), is shaped by sediment structure and particle size distribution [8]. Sediment plays a crucial role in defining the type, quantity, and availability of nutrients, which in turn sustain the primary producers at the base of the aquatic food web [8]. Micro-scale genetic structuring of *N. aperta* populations typically reflects reduced migration and increased population isolation, leading to distinct genetic patterns across different regions of the study area. Furthermore, several studies have employed ecological niche and habitat suitability models to support the control and management of waterborne disease transmission in Southeast Asia, including in Khong Island, Laos [8, 28].

Pairwise genetic differentiation (Φ_ST_) among the 13 localities revealed significant genetic structure based on *cox1* and 16S rRNA sequences, which is characteristic of genetic drift, a process in which random changes in allele frequencies drive divergence, particularly in small populations (Table 2). In addition, natural selection may also contribute to this structure by favoring different genetic variants in response to local environmental conditions. Together, these processes can lead to genetic isolation, resulting in inbreeding or adaptive divergence, where distinct snail populations evolve unique traits suited to their specific habitats. For example, a unique baseline of the biota, physical parameters, and water quality prior to dam construction has been described, providing a comparison of *N. aperta* habitats before and after dam construction, during the transmission of *S. mekongi* on the Mekong River [8]. The genetic structure of *N. aperta* populations was already differentiated based on their original groups, even before the construction of the dam [2, 27, 29]. This suggests that distinct genetic clusters existed between the populations, and the dam may have amplified or accentuated these differences. The pre-existing genetic differentiation could indicate that the populations were already somewhat isolated from one another or had adapted to different local environments [2]. The dam may have further restricted gene flow, leading to increased divergence between these original groups and potentially reinforcing the existing genetic differences observed in this study.

Alternatively, the distinct groups of *N. aperta* detected in this study may have been influenced by adaptation to specific local conditions, such as water temperature, substrate type, or food availability. These conditions could have created selective pressures that drove genetic differentiation [8]. The AMOVA results based on *cox1* and 16S rRNA sequences support the idea that *N. aperta* populations may have adapted to their local environments, leading to further divergence. If *N. aperta* populations remain isolated and experience inbreeding within populations, genetic diversity could decrease over time, potentially reducing the overall fitness of the populations. For example, the population genetic structure of *N. aperta* revealed numerous sub-populations, indicating limited large-scale dispersal and reducing the likelihood of cross-regional reinfection in the Mekong River [5]. The concept highlights that genetic differences within micro-scale distance populations of schistosomiasis parasites or hosts can influence the effectiveness of control measures. These variations are crucial when designing targeted interventions, as they can affect treatment success, parasite resistance, and transmission patterns. Tailoring control efforts to the genetic makeup of local populations ensures more efficient and precise management of schistosomiasis.

The presence of three strains (α, β, and γ) of *N. aperta* shows differences in physical characteristics, such as shell size and body pigmentation (Fig. 1), yet their genetic variation remains similar across these strains [1, 4]. This is an intriguing finding, suggesting that these morphological traits may be influenced more by environmental factors or selective pressures than by significant genetic divergence [1, 2, 5]. For instance, water quality plays a crucial role in the survival and reproduction of *N. aperta*—polluted or heavily disturbed water could reduce snail populations. Genetic differentiation among the strains could lead to the development of different ecological strategies, such as changes in reproductive timing, behavior, or tolerance to environmental stressors. These adaptations might help populations survive in their unique conditions, but also limit their ability to interact or breed with other populations [8]. Molecular markers, such as mitochondrial or nuclear DNA, could be useful for identifying specific genetic signatures of each group, helping to clarify the degree of divergence and potential evidence of local adaptation [2, 5]. However, our findings revealed data only from mitochondrial DNA. While this is valuable due to its high mutation rate and ease of amplification, its limitations mean it is best used in conjunction with nuclear markers. For comprehensive phylogeographic reconstructions in freshwater snails, incorporating data from both mitochondrial and nuclear DNA genomes offers a more accurate and balanced view of evolutionary history. For example, Paczesniak et al. [30] used both mitochondrial and nuclear DNA genetic data from a broad, geographically diverse sample to better understand the population structure of the freshwater snail *Potamopyrgus antipodarum*. This combined approach revealed insights into the evolution of sexual and asexual lineages that previous studies missed. Notably, they discovered unexpected nuclear discordance in asexual lineages, challenging assumptions about strict genome co-inheritance in these populations. This discordance may reflect past selection or rare instances of sexual reproduction in otherwise asexual populations.

Habitat can act as a barrier to gene flow, leading to genetic differentiation between populations occupying distinct ecological niches [5, 8, 28]. The high degree of habitat specialization observed in *N. aperta* supports the idea that environmental factors, such as water flow, substrate type, or predation pressure, play a significant role in shaping genetic structure. This local adaptation suggests that natural selection, alongside genetic drift, contributes to the observed patterns of divergence. For example, populations in fast-flowing rivers may evolve physiological or morphological traits that enhance attachment to substrates, while those in stagnant waters may adapt differently. These combined processes promote genetic isolation and the potential for adaptive divergence across the species’ range. As a result, the populations are becoming more genetically distinct from one another, with implications for host–parasite relationships and the potential for parasite adaptation [5, 8]. In terms of parasite control, this genetic structure could play an important role, as genetically distinct *N. aperta* populations with limited gene flow may harbor different parasite strains or exhibit varying levels of resistance to infection. As previous evidence revealed, the genetic differentiation of *N. aperta* depends on the different kinds of habitats, which low levels of migration between populations, suggesting well adaptations in snail sub-populations could influence disease transmission [5].

Evidence against the isolation-by-catchment hypothesis is provided by the haplotype network and AMOVA results, which revealed significant population structure among the ten different catchment systems, a pattern also observed in *Bithynia* snails [31]. The haplotype network revealed a few shared haplotypes, while the majority were exclusive to each catchment, representing the distinct genetic makeup of each region. This pattern suggests that *N. aperta* populations in the Mekong River are isolated by these catchments [1–5]. The presence of shared haplotypes between catchments can be attributed to snail dispersal between these geographically close localities, which are connected by floodplains [2, 5]. On the other hand, *N. aperta* is highly specific regarding water quality, preferring shallow areas with a pH greater than 7.5 [3], which may limit their dispersal to certain habitats. While genetic differentiation correlates with distinct habitat features, further functional or genomic studies are needed to confirm whether these differences are adaptive.

## Conclusions

Understanding the population genetics of *N. aperta* helps elucidate gene flow, migration patterns, and genetic differentiation between snail populations in different regions of the Mekong River basin. This knowledge is critical for identifying high-risk areas for schistosomiasis transmission and understanding the potential spread of the parasite across various environments. Modern sequencing technologies, including those used for nuclear DNA analysis, offer the opportunity to sequence the entire genome of *N. aperta*, enabling the identification of genetic markers associated with important traits such as heterozygosity and hybridization. Comparing the nuclear DNA of different strains (α, β, and γ) could reveal genetic differences linked to physical characteristics, such as shell size or pigmentation, as well as ecological adaptability, which may influence the survival and distribution of these populations. Thus, further investigation should focus on whole-genome comparisons among *N. aperta* strains to uncover candidate genes related to parasite susceptibility, environmental tolerance, and potential barriers to gene flow.

## Supplementary Information

Additional file 1. Supplementary Table 1. Sample localities of *Neotricula aperta* in the sub-catchments of the Mekong River based on *cox1* sequences.

## Data Availability

No datasets were generated or analyzed during the current study.

## Abbreviations

*cox1*: Cytochrome *c* oxidase subunit 1
16S rRNA: 16S ribosomal RNA
*N*: Number of *Neotricula aperta* examined
*s*: Number of polymorphic sites
*H*: Number of haplotypes
*Uh*: Unique haplotypes
Hd: Haplotype diversity
NJ: Neighbor-joining
ML: Maximum likelihood
MJN: Median-joining network
WHO: World Health Organization
Ss: Sum of squares
Vc: Variance components
PBCM: Population-based control model
