# Supplementary material for: Micro-scale genetic structure and genetic variation of Neotricula aperta (Gastropoda: Pomatiopsidae), the intermediate host of Schistosoma mekongi (Digenea: Schistosomatidae) in Champasak Province, Laos
Source: Trop Med Health. 2025 Jul 24;53:97. doi: 10.1186/s41182-025-00775-9 (PMC12288353; doi:10.1186/s41182-025-00775-9)
Supplement: Supplementary file 1 — Additional file 1. Supplementary Table 1. Sample localities of Neotricula aperta in the sub-catchments of the Mekong River based on cox1 sequences. [file 41182_2025_775_MOESM1_ESM.docx]

**Supplementary Table 1** Sample localities of *Neotricula aperta* in the sub-catchments of the Mekong River based on *cox1* sequences.

| **Code** | **Number** | **Strain** | **Village** | **District** | **Province** | **Catchment** | **Country** | **Latitude** | **Longitude** | **Date** | **Accession number** | **Reference** |
| --- | --- | --- | --- | --- | --- | --- | --- | --- | --- | --- | --- | --- |
| DKD02 | 4 | γ-strain | Don Kadent | Khong | Champasak | Huai Tomo | Laos | 14.038405 | 105.790263 | May-2024 | PV592087- PV592090 | This study |
| DK01 | 8 | γ-strain | Don Khone | Khong | Champasak | Huai Tomo | Laos | 13.964207 | 105.922658 | May-2024 | PV592091- PV592098 | This study |
| DK02 | 3 | γ-strain | Don Khone | Khong | Champasak | Huai Tomo | Laos | 13.968131 | 105.932354 | May-2024 | PV592099- PV592101 | This study |
| DK04 | 10 | γ-strain | Don Khone | Khong | Champasak | Huai Tomo | Laos | 13.959824 | 105.934357 | May-2024 | PV592102- PV592111 | This study |
| DK05 | 6 | γ-strain | Don Khone | Khong | Champasak | Huai Tomo | Laos | 13.955101 | 105.915562 | May-2024 | PV592112- PV592117 | This study |
| DK09 | 4 | γ-strain | Don Khone | Khong | Champasak | Huai Tomo | Laos | 13.969231 | 105.928031 | May-2024 | PV592118- PV592121 | This study |
| DK10 | 16 | γ-strain | Don Khone | Khong | Champasak | Huai Tomo | Laos | 13.960238 | 105.918434 | May-2024 | PV592122- PV592137 | This study |
| LK05 | 4 | γ-strain | Longkang | Khong | Champasak | Huai Tomo | Laos | 14.046277 | 105.801504 | May-2024 | PV592138- PV592141 | This study |
| LK14 | 2 | γ-strain | Longkang | Khong | Champasak | Huai Tomo | Laos | 14.044083 | 105.801917 | May-2024 | PV592142- PV592143 | This study |
| LK15 | 8 | γ-strain | Longkang | Khong | Champasak | Huai Tomo | Laos | 14.045833 | 105.801639 | May-2024 | PV592144- PV592151 | This study |
| SV08 | 6 | γ-strain | Somven-ok | Khong | Champasak | Huai Tomo | Laos | 14.007251 | 105.901286 | May-2024 | PV592152- PV592157 | This study |
| NLK03 | 7 | γ-strain | Nangloy Kang | Mounlapamok | Champasak | Huai Tomo | Laos | 14.434003 | 105.851324 | May-2024 | PV592158- PV592164 | This study |
| NLK04 | 2 | γ-strain | Nangloy Kang | Mounlapamok | Champasak | Huai Tomo | Laos | 14.432666 | 105.850273 | May-2024 | PV592165- PV592166 | This study |
| MOP | 21 | γ-strain | Mophou | Pathoumphon | Champasak | Huai Tomo | Laos | 14.985000 | 105.897000 | Apr-2001 | KU999563, KU999572, KU999690-KU999699, KU999706, KU999709, KU999715-KU999719, KU999722, KU999762 | Attwood et al. (2019) |
| HXK | 21 | γ-strain | Hat-Xai-Khoun | Pathoumphon | Champasak | Huai Tomo | Laos | 14.519000 | 105.870000 | Apr-2001 | KU999577-KU999582, KU999586, KU999591-KU999592, KU999595-KU999600, KU999603, KU999606-KU999608, KU999614, KU999656 | Attwood et al. (2019) |
| DKL | 14 | γ-strain | Don Kang Loi | Sukhuma | Champasak | Huai Tomo | Laos | 14.514000 | 105.859000 | Apr-2001 | KU999576, KU999583-KU999585, KU999587-KU999590, KU999593-KU999594, KU999601, KU999604, KU999613 | Attwood et al. (2019) |
| NFL | 10 | γ-strain | Na Fang | Khong | Champasak | Huai Tomo | Laos | 14.462000 | 105.861000 | Apr-2001 | KU999575, KU999602, KU999605, KU999609, KU999610-KU999611, KU999615-KU999618 | Attwood et al. (2019) |
| DNL | 8 | γ-strain | Don Nang Loi | Khong | Champasak | Huai Tomo | Laos | 14.373000 | 105.871000 | Apr-2001 | KU999574, KU999657-KU999663 | Attwood et al. (2019) |
| DPK | 6 | γ-strain | Don Phakan | Khong | Champasak | Huai Tomo | Laos | 13.926000 | 105.984000 | Apr-2001 | KU999570, KU999631-KU999632, KU999704, KU999713-KU999714 | Attwood et al. (2019) |
| DKX | 8 | γ-strain | Don Kaeng Xai | Samakkhixay | Attapeu | Se Kong | Laos | 14.811000 | 106.814000 | May-2003 | KU999531-KU999537, KU999666 | Attwood et al. (2019) |
| XKG | 10 | γ-strain | Attapeu | Samakkhixay | Attapeu | Se Kong | Laos | 14.785000 | 106.842000 | May-2003 | KU999538-KU999541, KU999545, KU999549-KU999551, KU999555, KU999558 | Attwood et al. (2019) |
| XKM | 12 | γ-strain | Kong-Phine | Samakkhixay | Attapeu | Se Kong | Laos | 14.771000 | 106.839000 | May-2003 | KU999542-KU999544, KU999546-KU999548, KU999552-KU999554, KU999556-KU999557, KU999559 | Attwood et al. (2019) |
| KLR | 20 | γ-strain | Kong Lor | Khounkham | Savanakhet | Nam Hinboun | Laos | 17.957000 | 104.732000 | Mar-2004 | KU999742-KU999761 | Attwood et al. (2019) |
| TKN | 19 | γ-strain | Thakhen | Hinboon | Khamouane | Nam Hinboun | Laos | 17.686000 | 104.695000 | Mar-2004 | KU999776-KU999794 | Attwood et al. (2019) |
| TOT | 19 | γ-strain | Thathot | Nhommalath | Khamouane | Se Bang Fai | Laos | 17.624000 | 105.145000 | Mar-2004 | KU999723-KU999741 | Attwood et al. (2019) |
| YOM | 6 | γ-strain | Yommarat | Nhommalath | Khamouane | Se Bang Fai | Laos | 17.604000 | 105.172000 | Apr-2001 | KU999681, KU999683, KU999768, KU999770, KU999772-KU999773 | Attwood et al. (2019) |
| MXL | 22 | γ-strain | Mahaxai | Mahaxay | Khamouane | Se Bang Fai | Laos | 17.413000 | 105.198000 | Apr-2001 | KU999670-KU999680, KU999682, KU999684, KU999763-KU999767, KU999769, KU999771, KU999774-KU999775 | Attwood et al. (2019) |
| BKV | 11 | γ-strain | Ban Kang Vang | Xaybuly | Khamouane | Se Bang Fai | Laos | 17.068000 | 105.070000 | Apr-2001 | MF999256-MF999266 | Attwood et al. (2019) |
| SST | 11 | γ-strain | Stung-Treng | Kaeh Sampeay | Stung-Treng | Siem Bok | Cambodia | 13.409000 | 105.940000 | Apr-2003 | KU999484-KU999486, KU999492-KU999493, KU999503, KU999512, KU999515, KU999523-KU999524, KU999528 | Attwood et al. (2019) |
| KSC | 17 | γ-strain | San Dan | Sanbour | Sambour | Siem Bok | Cambodia | 12.774000 | 105.963000 | May-2001 | KU999487-KU999491, KU999494-KU999497, KU999499-KU999500, KU999511, KU999522, KU999526, KU999529-KU999530, KU999795 | Attwood et al. (2019) |
| KRK | 20 | γ-strain | Krakor | Chetr Borei | Kratié | Siem Bok | Cambodia | 12.505000 | 106.014000 | May-2001 | KU999498, KU999501-KU999502, KU999504-KU999510, KU999513-KU999514, KU999516-KU999521, KU999525, KU999527 | Attwood et al. (2019) |
| SRG | 9 | γ-strain | Sri Goh | Sesan | Stung-Treng | Se San | Cambodia | 13.614000 | 106.375000 | Apr-2003 | KU999475-KU999483 | Attwood et al. (2019) |
| SDO | 11 | γ-strain | Sadao | Sesan | Stung-Treng | Se Kong | Cambodia | 13.610000 | 106.099000 | Apr-2003 | KU999636, KU999640, KU999643-KU999650, KU999655 | Attwood et al. (2019) |
| RAM | 18 | γ-strain | Krabi Chum | Sesan | Stung-Treng | Sre Pok | Cambodia | 13.553000 | 106.517000 | Apr-2003 | KU999396, KU999418-KU999424, KU999431, KU999433, KU999438-KU999440, KU999450, KU999545, KU999464, KU999466, KU999471 | Attwood et al. (2019) |
| OHG | 17 | γ-strain | Oh Gan | Koun Mom | Rattanakiri | Sre Pok | Cambodia | 13.490000 | 106.875000 | Apr-2004 | KU999393-KU999395, KU999404, KU999417, KU999427, KU999429, KU999437, KU999448-KU999449, KU999451, KU999456, KU999458-KU999461, KU999472 | Attwood et al. (2019) |
| DIL | 20 | γ-strain | Diloh | Lamphat | Rattanakiri | Sre Pok | Cambodia | 13.477000 | 107.008000 | Apr-2004 | KU999384, KU999387-KU999390, KU999406, KU999411, KU999432, KU999434, KU999442, KU999444-KU999447, KU999452, KU999455, KU999468-KU999470, KU999474 | Attwood et al. (2019) |
| TAL | 18 | γ-strain | Jua Talai | Lamphat | Rattanakiri | Sre Pok | Cambodia | 13.474000 | 106.996000 | Apr-2004 | KU999382-KU999383, KU999385-KU999386, KU999397-KU999400, KU999403, KU999405, KU999428, KU999441, KU999443, KU999453, KU999462-KU999463, KU999465, KU999473 | Attwood et al. (2019) |
| JND | 20 | γ-strain | Jua Negn Dai | Koun Mom | Rattanakiri | Sre Pok | Cambodia | 13.458000 | 106.877000 | Apr-2004 | KU999391-KU999392, KU999401-KU999402, KU999407-KU999410, KU999412-KU999416, KU999425-KU999426, KU999430, KU999435-KU999436, KU999457, KU999467 | Attwood et al. (2019) |
| BKL | 16 | γ-strain | Ban Khi-Lek | Khemmarat | Ubon Ratchathani | Se Bang Heang | Thailand | 16.036000 | 105.301000 | Apr-2001 | KU999560-KU999562, KU999564-KU999565, KU999567-KU999568, KU999573, KU999667-KU999669, KU999685-KU999689 | Attwood et al. (2019) |
| DAN | 20 | γ-strain | Ban Dan | Khong Chiam | Ubon Ratchathani | Mun | Thailand | 15.319000 | 105.504000 | May-2002 | KU999566, KU999569, KU999571, KU999619-KU999630, KU999633-KU999634, KU999702-KU999703, KU999707 | Attwood et al. (2019) |
| TK-NK | 6 | γ-strain | Ban Tha Kathin | Sri Chiang Mai | Nong Khai | Huai Luang | Thailand | Not given | Not given | Dec-2012 | KM099103-KM099108 | Limpanont et al. (2015) |
| KR-UB | 6 | γ-strain | Ban Bung Khi Lek | Khemmarat | Ubon Ratchathani | Mun | Thailand | Not given | Not given | Apr-2010 | KM099100-KM099102, KM099109-KM099111 | Limpanont et al. (2015) |
| KJ-UB | 6 | γ-strain | Khong Jeum | Khong Chiam | Ubon Ratchathani | Mun | Thailand | Not given | Not given | Apr-2010 | KM099096-KM099099, KM099112-KM099113 | Limpanont et al. (2015) |
| UB | 2 | β-strain | Kang Sapue | Phibun Mangsahan | Ubon Ratchathani | Mun | Thailand | Not given | Not given | Apr-2010 | KM099117-KM099118 | Limpanont et al. (2015) |
| UB | 3 | β-strain | Kang Sapue | Phibun Mangsahan | Ubon Ratchathani | Mun | Thailand | Not given | Not given | Nov-2012 | KM099119-KM099121 | Limpanont et al. (2015) |
| UB | 3 | β-strain | Kang Sapue | Phibun Mangsahan | Ubon Ratchathani | Mun | Thailand | Not given | Not given | Nov-2010 | KM099114-KM099116 | Limpanont et al. (2015) |
| LGAM | 2 | γ-strain | Ban Xieng-Wang | Khong | Champasak | Huai Tomo | Laos | 14.630000 | 105.514500 | Apr-1997 | AF188220-AF188221 | Attwood et al. (2001) |
| XBFG | 1 | γ-strain | Ban Mahaxai | Xebangfay | Khamouane | Se Bang Fai | Laos | 17.244500 | 105.131500 | Mar-1998 | AF188227 | Attwood et al. (2001) |
| KHMR | 3 | γ-strain | Krakor | Chetr Borei | Kratié | Siem Bok | Cambodia | 12.271000 | 106.145000 | Jun-1996 | AF188217-AF188219 | Attwood et al. (2001) |
| TGAM | 5 | γ-strain | Ban Khi-Lek | Khemmarat | Ubon Ratchathani | Mun | Thailand | 16.064334 | 105.160993 | Apr-1997 | AF188222-AF188226 | Attwood et al. (2001) |
| BETA | 4 | β-strain | Kaeng-Kao | Phibun Mangsahan | Ubon Ratchathani | Mun | Thailand | 15.143000 | 105.171700 | Apr-1997 | AF188213-AF188216 | Attwood et al. (2001) |
| ALPH | 3 | α-strain | Ban Khi-Lek | Khemmarat | Ubon Ratchathani | Mun | Thailand | 16.064334 | 105.160993 | Apr-1997 | AF188210-AF188212 | Attwood et al. (2001) |
| **Total** | **528** |  |  |  |  |  |  |  |  |  |  |  |
